# SIRT7-Induced PHF5A Decrotonylation Regulates Aging Progress Through Alternative Splicing-Mediated Downregulation of CDK2

**DOI:** 10.3389/fcell.2021.710479

**Published:** 2021-09-17

**Authors:** Ai Qing Yu, Jie Wang, Shi Tao Jiang, Li Qun Yuan, Hai Yan Ma, Yi Min Hu, Xing Min Han, Li Ming Tan, Zhi Xiao Wang

**Affiliations:** ^1^Department of Clinical Laboratory, Hunan Provincial People’s Hospital (The First-Affiliated Hospital of Hunan Normal University), Changsha, China; ^2^Department of Cardiology, Taihe Hospital, Hubei University of Medicine, Shiyan, China; ^3^Department of Nuclear Medicine, The First Affiliated Hospital of Zhengzhou University, Zhenzhou, China; ^4^Department of Cardiology, The Fifth Affiliated Hospital of Zunyi Medical University, Zhuhai, China

**Keywords:** Sirt7, PHF5A, crotonylation, cellular aging, alternative splicing

## Abstract

Dysregulation of protein posttranslational modification (PTM) can lead to a variety of pathological processes, such as abnormal sperm development, malignant tumorigenesis, depression, and aging process. SIRT7 is a NAD^+^-dependent protein deacetylase. Besides known deacetylation, SIRT7 may also have the capacity to remove other acylation. However, the roles of SIRT7-induced other deacylation in aging are still largely unknown. Here, we found that the expression of SIRT7 was significantly increased in senescent fibroblasts and aged tissues. Knockdown or overexpression of SIRT7 can inhibit or promote fibroblast senescence. Knockdown of SIRT7 led to increased pan-lysine crotonylation (Kcr) levels in senescent fibroblasts. Using modern mass spectrometry (MS) technology, we identified 5,149 Kcr sites across 1,541 proteins in senescent fibroblasts, and providing the largest crotonylome dataset to date in senescent cells. Specifically, among the identified proteins, we found SIRT7 decrotonylated PHF5A, an alternative splicing (AS) factor, at K25. Decrotonylation of PHF5A K25 contributed to decreased CDK2 expression by retained intron (RI)-induced abnormal AS, thereby accelerating fibroblast senescence, and supporting a key role of PHF5A K25 decrotonylation in aging. Collectively, our data revealed the molecular mechanism of SIRT7-induced k25 decrotonylation of PHF5A regulating aging and provide new ideas and molecular targets for drug intervention in cellular aging and the treatment of aging-related diseases, and indicating that protein crotonylation has important implications in the regulation of aging progress.

## Introduction

In eukaryotic cells, protein posttranslational modification (PTM) achieves rapid functional adaptation to various intracellular and external signals by regulating enzyme activity, and protein stability. Abnormal PTM can lead to various pathological conditions, such as sperm development defects, malignant transformation, and depression (Liu et al., 2017, 2019; Yu H. et al., 2020). With the development of modern mass spectrometry (MS) technology, a series of short-chain lysine acylation modifications including lactylation, crotonylation, β-hydroxyisobutyrylation, succinylation, and other new acylation modifications have been discovered (Sabari et al., 2017; Zhang et al., 2019). These novel acylation modifications are similar to the well-known lysine acetylation modification structure. However, the functions of these newly discovered acylation modifications still need to be elucidated.

As a member of the silent information regulator-2 (Sir2) protein family, sirtuins are NAD^+^-dependent protein deacetylases or ADP-ribosyltransferases, and are highly conserved from prokaryotes to eukaryotes (Wu et al., 2018). In mammals, the sirtuin family includes seven Sir2 homologous proteins (SIRT1–7), which share a conserved NAD^+^-dependent catalytic core domain, located in different subcellular compartments, targeting different substrates and enzyme activity, and control various important biological processes, including energy metabolism, resistance to stress, genome stability, aging, and tumors (Kida and Goligorsky, 2016). In addition to its deacetylase function to regulate DNA damage and repair, and SIRT7 also has a desuccinylation function to regulate genome stability (Li et al., 2016). Various studies have linked the functions of the sirtuin family to cellular senescence and lifespan extension and attributed these functions to SIRT1 and SIRT6 (Wa̧troba et al., 2017). Recently, researchers revealed that SIRT3 interacts with nuclear fibrils and heterochromatin proteins to maintain the stability of heterochromatin and regulate cellular senescence (Diao et al., 2021). In addition, studies have found that the nucleolar protein nucleophosmin (NPM1) is deacetylated by SIRT7 to affect cellular senescence (Lee et al., 2014). So, can SIRT7 mediate other deacylation modifications to regulate aging?

Alternative splicing (AS) is one of the crucial posttranscriptional regulatory mechanisms that regulate the translation of mRNA subtypes and produce protein diversity (Zong et al., 2018). Splicing is carried out through spliceosome, which is a huge complex consisting of hundreds of proteins and five small nuclear ribonucleoproteins (snRNP) named U1, U2, U4, U5, and U6 (Matera and Wang, 2014). PHF5A is an important AS factor, which encodes a subunit of splicing factor 3b protein complex (Zheng et al., 2018). PHF5A participates in the formation of spliceosome together with splicing factors 3b, 3a, and 12S RNA and maintain the stability of SF3b spliceosome (Wang et al., 2019). As a component of U2snRNPs, PHF5A is a scaffold protein that interacts with SF3B1, SF3B3, SF3B5, and intron pre-mRNA. SF3B1 directly cross-links with nucleotides upstream and downstream, while PHF5A acts as a chaperone in branch point sequence (BPS) recognition (Hoskins and Moore, 2012). The SF3B complex recognizes BPS and forms a splicing precursor, and then completes the pre-mRNA splicing process, thereby regulating the AS expression of genes (Wang et al., 2019).

In the present study, we used a quantitative proteomics approach to gain a global understanding of the crotonylome alterations in response to SIRT7 knockdown (SIRT7KD) in senescent fibroblasts. We identified 1,468 Kcr sites across 1,541 proteins in senescent fibroblasts, providing by far the largest crotonylome dataset in senescent cells. Specifically, we found SIRT7 decrotonylated PHF5A at K25. PHF5A K25 decrotonylation contributed to decreased CDK2 expression by inducing abnormal AS, which accelerated fibroblast senescence.

## Results

### SIRT7 Expression Is Significantly Increased in Senescent Fibroblasts and Aged Tissues

**FIGURE 1:**
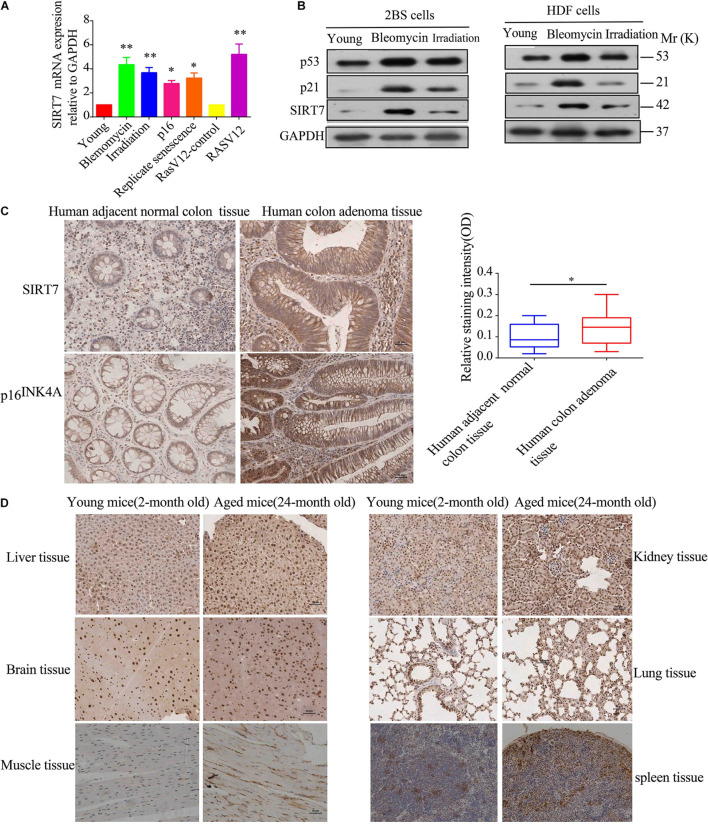
SIRT7 expression in senescent fibroblasts and aged tissues. **(A)** The mRNA expression level of SIRT7 in various senescent cells, young cells, or control cells; the experiments were repeated three times, ^∗^*P* < 0.05, ^∗∗^*P* < 0.01. **(B)** The protein expression level of SIRT7 in various senescent cells and young cells; the experiments were repeated three times. **(C)** SIRT7 expression in human colon adenoma and human adjacent normal colon tissue, *n* = 10, statistical analysis by *t*-test, ^∗^*p* < 0.05. Scale bar = 30 μm. **(D)** Expression of SIRT7 in various tissues of naturally aged mice and young mice, *n* = 3. Scale bar = 30 μm.

Firstly, we constructed young fibroblasts [below population doublings (PD)28], replicative senescent fibroblasts (PD 62), and bleomycin, irradiation, p16, and Ras V12-induced premature senescence. Then, we detected the relative expression levels of SIRT7 mRNA and protein in young fibroblasts, replicative senescent fibroblasts, and premature senescent fibroblasts. We found that the expression levels of SIRT7 mRNA and protein were significantly elevated in replicative senescent fibroblasts, and premature senescent fibroblasts compared to young fibroblasts or corresponding control groups (Figures 1A,B). Secondly, we employed immunohistochemistry (IHC) to detect SIRT7 expression in 24-month-old naturally aged male mice and human colon adenoma tissues, and two commonly used and widely accepted aged models (identified by p16^INK4A^ staining and SA-gal staining) (Yang et al., 2017; Yu A.Q. et al., 2020). Our data show that SIRT7 expression is obviously increased in human colon adenoma tissues (Figure 1C) and naturally aged male mice tissues (Figure 1D) in comparison to corresponding control groups. Taken together, these data indicated that SIRT7 expression is significantly increased in senescent cells and aged tissues.

### SIRT7 Promotes Fibroblast Senescence

**FIGURE 2:**
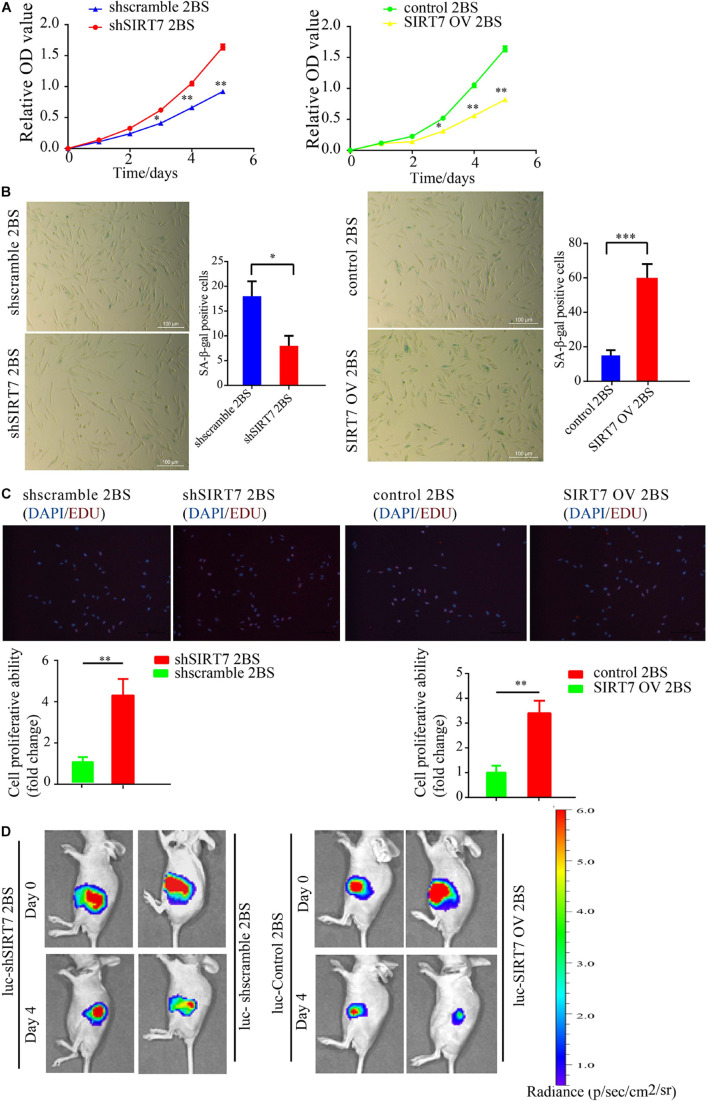
The effect of SIRT7 on 2BS fibroblast senescence. **(A)** CCK8 evaluated the proliferative ability of 2BS fibroblasts with SIRT7 knockdown or overexpression (OV); the experiments were repeated three times. **(B)** SA-β-gal staining of 2BS fibroblasts with SIRT7 knockdown or overexpression, scale bar = 100 μm; the experiments were repeated three times, ^∗∗^*P* < 0.01. **(C)** EdU incorporation analysis of proliferative ability of SIRT7 knockdown or overexpression 2BS fibroblasts, scale bar = 200 μm; the experiments were repeated three times. **(D)** The VIS spectrum imaging system was used to analyze the luciferase activity 4 days after SIRT7 knockdown or overexpression 2BS fibroblast transplantation. All experiments were repeated three times independently, and statistical analysis was performed by *t*-test, ^∗^*p* < 0.05, ^∗∗^*p* < 0.01, and ^∗∗∗^*p* < 0.001.

**FIGURE 3:**
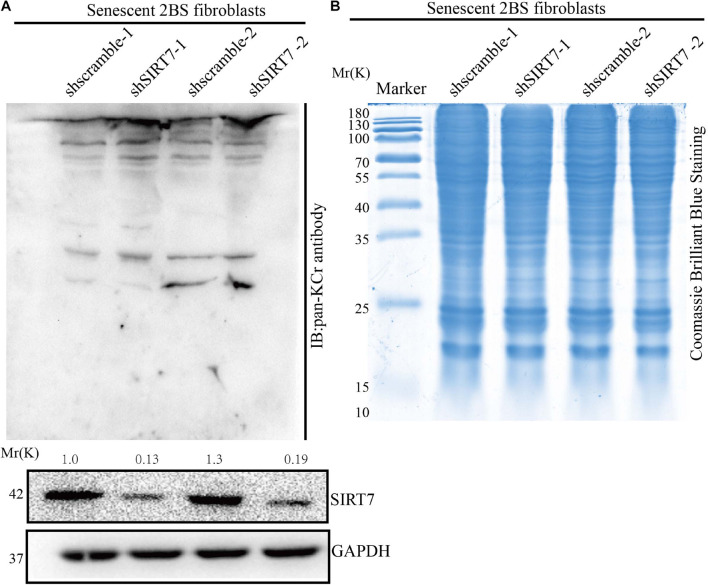
Knockdown of SIRT7 altered pan-crotonylation level in senescent fibroblasts. **(A)** Pan-Crotonylation modification antibody was used to detect the pan-crotonylation level in shscramble and shSIRT7 senescent 2BS fibroblasts. **(B)** Coomassie brilliant blue staining of total protein of shscramble and shSIRT7 senescence 2BS fibroblast. All experiments were repeated three times independently.

**FIGURE 4:**
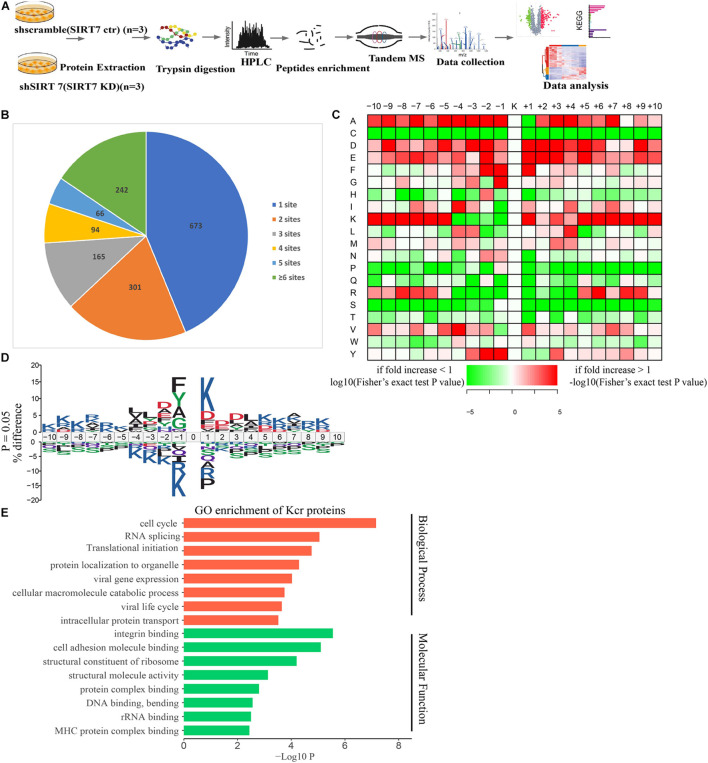
Profiling Kcr proteome in senescence 2BS fibroblast. **(A)** Schematic diagram of the experimental workflow for identification of protein Kcr in WT and SIRT7 KD senescence 2BS fibroblast. **(B)** Pie chart showing the distribution of the number of identified Kcr sites per protein. **(C)** Motif analysis of all identified Kcr proteins. **(D)** IceLogo representation showing flanking sequence preferences for all Kcr sites. **(E)** Bar graphs showing representative ontology annotations enriched with Kcr proteome.

Before exploring the effects of SIRT7 on fibroblast senescence, we constructed sh-SIRT7 (SIRTKD) or SIRT7 overexpression (OV) 2BS fibroblasts by transfecting lentivirus expressing shSIRT7 or SIRT7 OV. We found shscramble and SIRT7 OV 2BS fibroblasts exhibiting a series of premature phenotypes compared to shSIRT7, and SIRT7 control (ctr), respectively, including (1) a slower CCK8 growth curve (Figure 2A), (2) increased levels of senescence-associated–β-galactosidase (SA-β-gal) activity (Figure 2B), (3) decreased percentage of EDU-positive cells (Figure 2C), and (4) shSIRT7, shscramble, SIRT7 OV, and SIRT7 ctr 2BS fibroblasts transfected with a lentiviral vector expressing luciferase (Luc) and injected into the abdominal subcutaneous of immunodeficient mice. Consistent with the *in vitro* observations, shscramble and SIRT7 OV 2BS fibroblasts exhibited an accelerated functional decay after transplantation *in vivo* (Figure 2D).

### Knockdown of SIRT7 Leads to Increased Pan-Kcr Levels in Senescent Fibroblasts

Besides known deacetylation, SIRT7 may also have the capacity to remove other acylation, such as desuccinylation. Here we reason that SIRT7 could function as a de-crotonylase in senescent fibroblasts. To measure the alteration of pan-Kcr levels in SIRTKD senescent fibroblasts, we employed the pan-Kcr antibody to detect the pan-Kcr levels in SIRTKD-senescent fibroblasts. Here we made a postulation that SIRT7 could function as a de-crotonylase in senescent fibroblasts (Figures 3A,B). Our data preliminarily suggested that SIRT7 induced decrotonylation, thereby triggering elevated levels of pan-Kcr in senescent fibroblasts. These data indicate that beside playing a role as a histone deacetylase (HDAC), SIRT7 could function as a decrotonylase.

### Global Landscape of SIRT7-Regulated Crotonylome in Senescent Fibroblasts

To obtain a comprehensive view of SIRT7-regulated crotonylome, especially Kcr of nonhistone substrates, we used an integrated approach involving TMT/iTRAQ (tandem mass tag/isobaric tagging for multiplexed relative and absolute protein quantitation) labeling, HPLC (high-performance liquid chromatography) fractionation, immunoaffinity enrichment, and high-resolution LC-MS/MS (liquid chromatography tandem MS) to profile Kcr substrates upon SIRT7 KD in senescent fibroblasts. A total of 6 × 10^7^ shSIRT7 and shscramble senescent fibroblasts were harvested, and lysed. After trypsin digestion, peptides were separated into 12 fractions by high-pH reversed-phase HPLC, and Kcr-containing peptides were enriched with immobilized anti-Kcr and analyzed by LC-MS/MS (Figure 4A). A total of 5,149 Kcr sites across 1,541 proteins were identified, with 4,267 Kcr sites from 1,298 proteins quantified. Among these Kcr proteins, 673 (51.8%) had a single Kcr site, and 301 (23.2%) had more than six Kcr sites (Figure 4B).

We next evaluated the amino acid flanking of the identified Kcr sites against all human background sequences using iceLogo software. Obvious enrichment of negatively charged aspartic acid was found at −2 and +2 of Kcr sites (Figures 4C,D). Gene ontology (GO) analysis of potential Kcr-regulated intracellular pathways revealed that upregulated Kcr proteins are associated with diverse biological processes including cell cycle, RNA splicing, translation initiation, and DNA/protein binding (Figure 4E).

### Quantitative Analysis of Kcr Proteome in SIRT7 KD Senescent Fibroblasts

**FIGURE 5:**
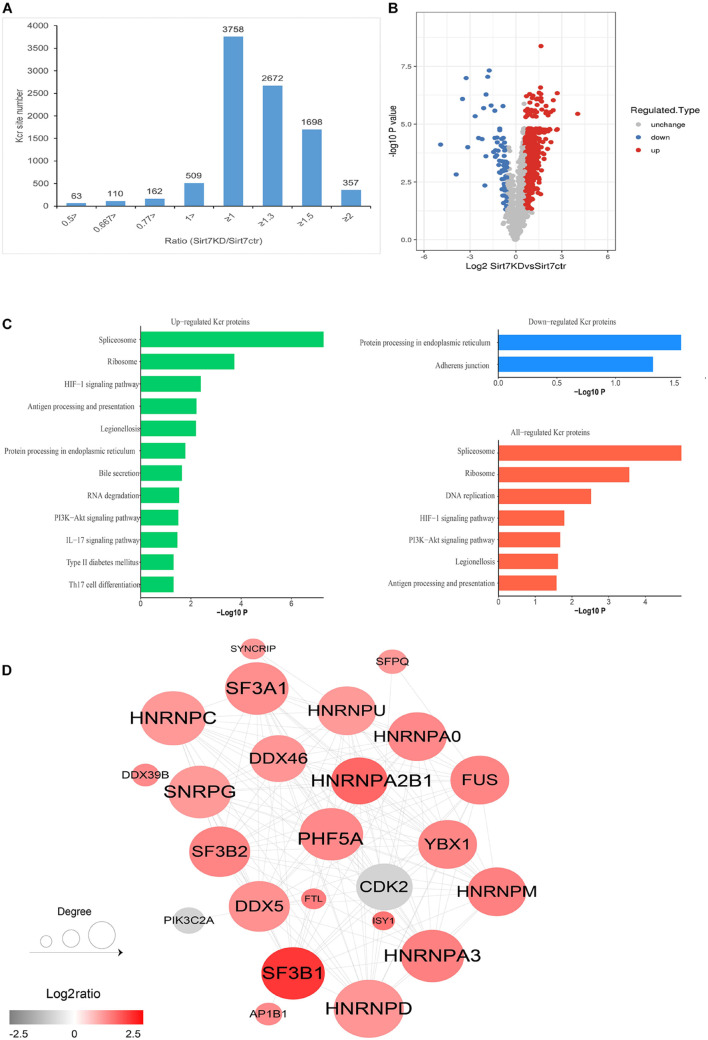
Quantification analysis of crotonylome in response to SIRT7 KD. **(A)** Histogram showing the ratio distribution of quantifiable Kcr sites between WT and SIRT7 KD senescence 2BS fibroblast. **(B)** Volcano plot showing the differential expression profile of Kcr protein in WT and SIRT7 KD senescence 2BS fibroblast. **(C)** Bar graphs showing the KEGG pathway associated with all identified, upregulated, and downregulated Kcr proteins. **(D)** Protein–protein interaction network of SIRT7-regulated Kcr proteome based on the STRING database.

We next quantified the alteration of protein Kcr upon SIRT7 KD in comparison to total protein abundance in senescent fibroblasts. The cutoff ratio for statistically significant Kcr changes between SIRT7 KD and WT senescent fibroblasts was set to above 1.5 or below 0.67. The results showed that 1468 Kcr sites in 676 proteins were upregulated, and 80 Kcr sites in 74 proteins were downregulated in SIRT7 KD senescent fibroblasts (Figures 5A,B). KEGG (Kyoto Encyclopedia of Genes and Genomes) analysis revealed that upregulated Kcr proteins are mainly involved in spliceosome, ribosome, and DNA replication, while downregulated Kcr proteins are enriched in pathways associated with protein processing in endoplasmic reticulum and adherens junction (Figure 5C). Using MCODE (Minimal Common Oncology Data Elements), several highly associated Kcr protein subnetworks of Kcr proteins were identified from SIRT7-regulated Kcr proteome, mainly including spliceosome-related protein PHF5A and SF3Bs, and cell-cycle regulator CDK2, etc (Figure 5D).

### SIRT7 Decrotonylated PHF5A at K25

**FIGURE 6:**
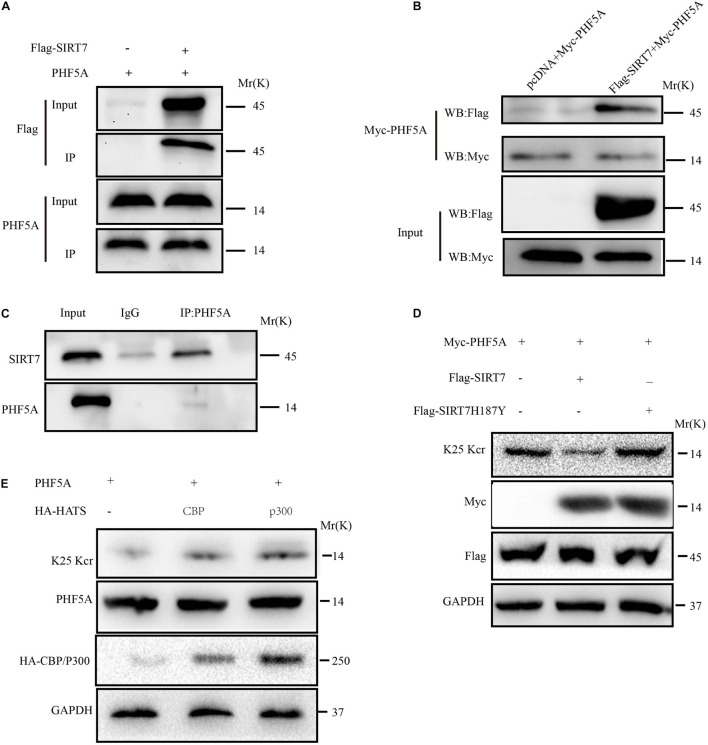
SIRT7 decrotonylated PHF5A at K25. **(A)** Semi-exogenous Co-IP confirmed that SIRT7 has an exogenous interaction with PHF5A. Semi-exogenous Co-IP detected the interaction between exogenous SIRT7 and endogenous PHF5A in 2BS fibroblast transduced with lentiviruses expressing Flag-SIRT7. **(B)** Exogenous Co-IP confirmed that SIRT7 has an exogenous interaction with PHF5A. Exogenous Co-IP detected the interaction between exogenous SIRT7 and exogenous PHF5A in 2BS fibroblast transduced with lentiviruses expressing Flag-SIRT7, and myc-PHF5A, respectively. **(C)** Endogenous Co-IP confirms that SIRT7 and PHF5A have an endogenous interaction. **(D)** The PHF5A K25 site-specific Kcr antibody confirmed SIRT7-mediated decrotonylation of PHF5A at K25. **(E)** CBP/p300 increased crotonylation of PHF5A at K25; HEK293T cells were transfected with plasmids as indicated, and indicated proteins were immunoblotted by the corresponding antibody. All experiments were repeated three times independently.

Among the upregulated Kcr proteins, PHF5A strongly attracted the authors’ interest. The Kcr level of PHF5A k25 in the SIRT7 KD senescent fibroblasts was increased by 2.869 times compared with shscramble senescent fibroblasts (Supplementary Figure 1A). MS identified a crotonylation site of PHF5A at k25 (Supplementary Figure 1B). Conservation analysis of PHF5A indicated that K25 is a highly conserved site from *Equus caballus* to Homo sapiens (Supplementary Figure 1C). Therefore, we preliminarily reasoned that PHF5A is the direct targets of SIRT7. Next, immunoprecipitation (IP) and GST pull-down were performed to confirm the *in vivo* and *in vitro* interactions. Our data revealed that PHF5A interacted with SIRT7 *in vivo* and *in vitro* (Figures 6A–C). To further confirm that SIRT7 decrotonylated PHF5A at K25, we generated a PHF5A K25 site-specific Kcr antibody (termed as PHF5A K25 Kcr). Myc-PHF5A, Flag-SIRT7, and Flag-SIRT7H187Y [substitution of the highly conserved histidine residue (His187) in the predicted catalytic domain with tyrosine] were co-transfected into young 2BS fibroblasts. The Kcr level of PHF5A K25 was analyzed by Western blot with antibodies against PHF5A K25 Kcr. Consistent with MS results, our data revealed that SIRT7 induced decrotonylation of PHF5A at K25, which led to reduced Kcr levels of PHF5A K25 in young 2BS fibroblasts (Figure 6D). Previously published research uncovered that members of the acetyltransferase superfamily may function as crotonyltransferases to catalyze the crotonylation of nonhistone proteins, mainly including CBP, P300, PCAF, and hMOF (Xu et al., 2017). In this research, we also found that CBP and p300 are mainly crotonyltransferases in fibroblasts, suggesting that the acetyltransferases CBP/p300 also act as crotonyltransferases for nonhistone proteins in fibroblasts (Figure 6E).

### PHF5A K25 Decrotonylation Accelerate Fibroblasts Senescence by AS-Mediated Downregulation of CDK2

**FIGURE 7:**
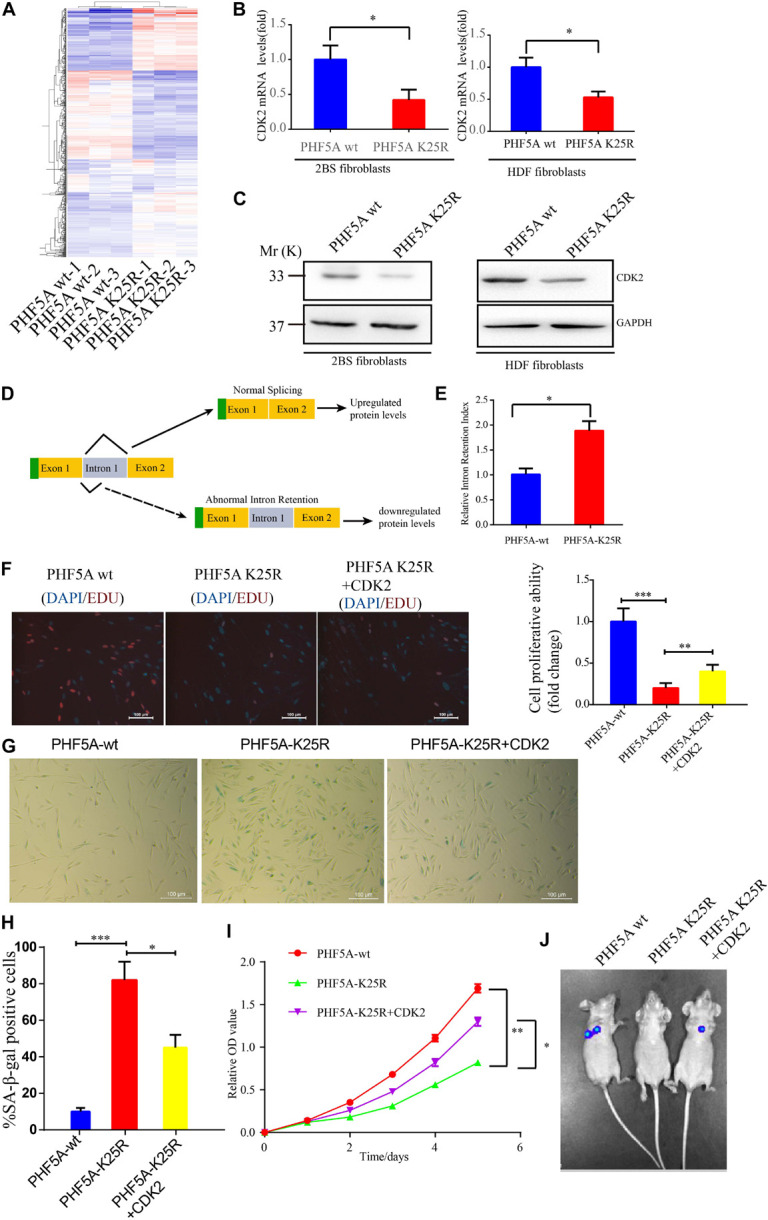
PHF5A K25 decrotonylation accelerates fibroblast senescence by alternative splicing (AS)-mediated downregulation of CDK2. **(A)** Heat map of differentially expressed genes from RNA-sequencing analysis between PHF5A-WT and PHF5A-K29R cells. PHF5AK29R downregulates CDK2 mRNA and protein levels. **(B,C)** Quantitative real-time PCR analysis (*n* = 3) **(B)** and immunoblotting analysis of CDK2 in 2BS fibroblast **(C)**. **(D)** Schematics of the CDK2 (NM_001798.5) alternative splicing pattern regulated by decrotonylated PHF5A. Full line and dotted line represent normal splicing and abnormal intron retention, respectively. Blue and yellow boxes represent the untranslated region (UTR) and protein-coding region (CDS), respectively. **(E)** PHF5A K29R increased the CDK2 splicing index for intron 1 retention. Quantitative real-time PCR analysis of normal or abnormal splicing isoforms of CDK2 (*n* = 3). **(F)** EdU incorporation analysis of the proliferative ability of PHF5Awt, PHF5A K25R, and PHF5A K25R+CDK2 2BS fibroblasts, scale bar = 100 μm. **(G,H)** SA-β-gal staining analysis of PHF5Awt, PHF5A K25R, and PHF5A K25R+CDK2 2BS fibroblasts, scale bar = 100 μm. **(I)** CCK8 analysis of the proliferative ability of PHF5Awt, PHF5A K25R, and PHF5A K25R+CDK2 2BS fibroblasts. **(J)** The IVIS spectrum imaging system was used to analyze the luciferase activity 4 days after transplantation. All experiments were repeated three times independently, and statistical analysis was performed by *t*-test, ^∗^*p* < 0.05, ^∗∗^*p* < 0.01, and ^∗∗∗^*p* < 0.01.

Alternative splicing we known, PHF5A is involved in AS regulation for transcription and pre-mRNA splicing. We investigated whether acetylation of PHF5A participates in the regulation of these processes. The RNA-sequencing analysis was performed to identify changes in gene expression and AS in PHF5A-WT, and PHF5A-K25R (lysine to arginine mutant for protein-decrotonylated state mimic). A previous study has reported that acetylation of PHF5A at K29 increased KDM3A gene expression by RI-induced regulation of KDM3A pre-mRNA AS (Wang et al., 2019). The RNA-sequencing data indicated that CDK2 mRNA levels were dramatically reduced in PHF5A K25R compared to PHF5A wt (Figure 7A and Supplementary Table 1). Combining transcriptome sequencing data with splicing bioinformatics analysis, we found the differentially expressed gene group and the differentially splicing gene group induced by RI AS type (Supplementary Table 2). CDK2, one of these differentially splicing genes, immediately attracted our attention. As a serine/threonine protein kinase, CDK2 functions as a regulator of cell-cycle regulation. Consistent with the RNA-sequencing analysis by Leafcutter, real-time qPCR and immunoblot confirmed that CDK2 mRNA, and protein levels were significantly decreased in PHF5A K25R compared to PHF5A wt (Figures 7B,C). We further revealed that PHF5A K25 decrotonylation increased the abnormal efficiency of CDK2 pre-mRNA exon 1/intron 1 splicing (Figures 7D,E).

Furthermore, young 2BS fibroblasts with PHF5A K25R OV trigger a series of premature phenotypes compared to PHF5A wt young 2BS fibroblasts, including (1) decreased percentage of EDU-positive cells (Figure 7F), (2) increased levels of SA-β-gal activity (Figures 7G,H), (3) slower CCK8 growth curve (Figure 7I), and (4) accelerated functional decay after transplantation *in vivo* (Figure 7J), which could be rescued by overexpressing CDK2 in young 2BS fibroblasts with PHF5A K25R OV. Taken together, our data indicated that SIERT7-induced PHF5A K25 decrotonylation promotes fibroblast senescence by reducing CDK2 expression through abnormal AS.

## Discussion

Dynamic and versatile protein PTMs are an important way to induce the ever-changing extracellular signals to intracellular effects. Particularly, in eukaryotic cells, reversible histone PTMs are important in regulating gene expression to quickly adapt to changes in the internal and external environment of the cell (Liu et al., 2019). Based on the development of MS technology, a series of short-chain lysine acylation modifications including lactylation, crotonylation, β-hydroxyisobutyrylation, and succinylation have been identified. Although these newly identified acylation is structurally similar to the well-known lysine acetylation, their function in the regulation of gene expression and disease is distinctive from acetylation. Novel acetylation of histones including lactylation, crotonylation, β-hydroxyisobutyrylation, and succinylation can lead to various pathophysiological processes by regulating gene expression. Histone acetylation and methylation are the most common chromatin remodeling factors that regulate aging. A series of elegant studies have revealed that histone methylation markers such as H3K4me3, H3K9me3, H3K27me3, and H3K36me3 and histone acetylation markers such as H3K9ac, H3K56ac, H4K12ac, and 4K16ac are involved in the regulation of aging (Yi and Kim, 2020).

However, there are few studies on the regulation of aging by nonhistone PTM, especially nonhistone new acylation modification. In this study, we identified a series of histone and nonhistone Kcr in senescent fibroblasts. We identified 5,149 Kcr sites from 1,541 proteins in senescent fibroblasts, providing the largest crotonylome dataset to date in senescent cells. Crotonylated proteins were mainly distributed in the cytoplasm (39%), the nucleus (25%) and the mitochondria (13%). Yu H. et al. (2020) also identified 14,311 Kcr sites from 3,734 proteins in HeLa cells, providing by far the largest crotonylome dataset in tumor cells. Compared with tumor cells, senescent fibroblasts have a lower abundance of crotonylome.

Among SIRT7-regulated Kcr proteins, PHF5A is an important splicing factor essential for the pre-mRNA splicing process. We found that SIRT7-induced PHF5A K25 decrotonylation influenced spliceosome activity by altering the stability of the U2 snRNP complex. PHF5A K25R undermined the interaction between PHF5A and SF3Bs, thereby impairing spliceosome activity compared to PHF5A wt in senescent fibroblasts. As stated earlier, it has been reported that PHF5A was acetylated at k29 to modulate stress responses, and colorectal carcinogenesis (Wang et al., 2019). In parallel, we revealed that PHF5A was crotonylated at k25 by SIRT7 to regulate aging, suggesting that PHF5A triggers different PTMs at different lysine sites to confront various pathophysiological process. So far, only one acetylation site (k29) and one crotonylation site (k25) have been found on PHF5A. However, whether these two modifications will coexist and regulate the aging process in a synergistic manner needs further research.

An earlier study described that SIRT7 was first known for its deacetylase function. In 2016, Li et al. (2016) found that SIRT7 can also function as a histone desuccinylase. In this study, we revealed that SIRT7 can also function as a histone and nonhistone decrotonylase, indicating that SIRT7 could perform different functions through different deacylation modifications to adapt to various intracellular, and external signals by regulating enzyme activity and protein stability.

Although we uncovered that PHF5A K25R led to abnormal AS of CDK2, there is no evidence to show which type of AS induced the abnormal AS of CDK2. Currently, there are seven splicing modes of AS: ① Alternate acceptor (AA), ② Alternate donor (AD), ② Alternate Promoter (AP), ③ Alternate terminator (AT), ④ Exon skip (ES), ⑤ Mutually exclusive exon (ME), and ⑥ Retained intron (RI) (Teng et al., 2017). Wang et al., revealed that PHF5A k29 acetylation induced AS-mediated upregulation of KDM3A through the RI AS type. In this study, PHF5A k25 decrotonylation also triggered the RI AS type to induced AS-mediated downregulation of CDK2, indicating that PHF5A acylation regulates downstream target gene expression mainly through RI-induced AS. However, the mechanism of PHF5A acylation (K29 acetylation or K25 crotonylation) affecting AS needs further investigation. One of the main reasons, we think, is that PHF5A acylation (K29 acetylation or K25 crotonylation) affects the interaction with SF3B1, SF3B2, SF3B3, SF3B4, and SF3B5, which influence the stability of spliceosome, and thereby affecting AS.

**FIGURE 8:**
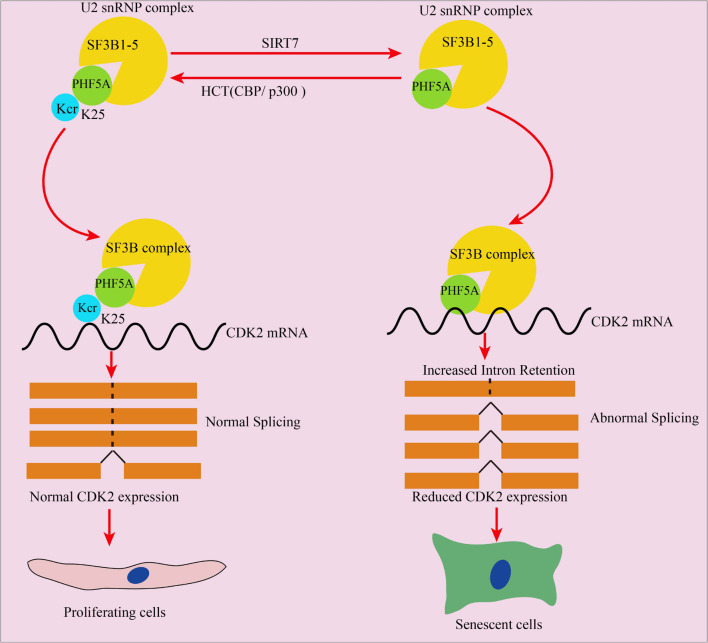
Schematic illustration of PHF5A decrotonylation at K25 by SIRT7 regulates aging progress through alternative splicing mediated downregulation of CDK2. PHF5A could be decrotonylated and crotonylated at K25 by SIRT7 and CBP/p300, respectively. Decrotonylation of PHF5A at K25 reduced downstream target CDK2 mRNA and protein expression through RI-induced alternative splicing, and thereby accelerating cellular aging.

Overall, this manuscript revealed that decrotonylation of PHF5A, a key protein among spliceosomes, and acts as a responder during fibroblast aging progress. It reduced downstream target CDK2 mRNA and protein expression through RI-induced AS, thereby accelerating cellular aging (Figure 8). These findings expand the field of novel protein PTM regulation especially nonhistone protein in cellular aging and provide a potential target for slowing down aging.

## Materials and Methods

### Identification of Acetylation Site by LC-MS/MS Analysis

#### Trypsin Digestion

For digestion, the protein solution was reduced with 5 mM dithiothreitol for 30 min at 56°C and alkylated with 11 mM iodoacetamide for 15 min at room temperature in darkness. The protein sample was then diluted by adding 100 mM TEAB to a urea concentration less than 2 M. Finally, trypsin was added at a 1:50 trypsin-to-protein mass ratio for the first digestion overnight and a 1:100 trypsin-to-protein mass ratio for a second 4-h digestion.

#### TMT/iTRAQ Labeling

After trypsin digestion, the peptide was desalted using the Strata-X C18 SPE column (Phenomenex, Torrance, CA, United States), and vacuum-dried. The peptide was reconstituted in 0.5 M TEAB and processed according to the manufacturer’s protocol for TMT kit/iTRAQ kit. Briefly, one unit of TMT/iTRAQ reagent was thawed and reconstituted in acetonitrile. The peptide mixtures were then incubated for 2 h at room temperature and pooled, desalted, and dried by vacuum centrifugation.

#### HPLC Fractionation

The tryptic peptides were fractionated into fractions by high-pH reverse-phase HPLC using a Thermo BetaSil C18 column (5-μm particles, 10 mm ID, and 250 mm length). Briefly, peptides were first separated with a gradient of 8 to 32% acetonitrile (pH 9.0) over 60 min into 60 fractions. Then, the peptides were combined into six fractions and dried by vacuum centrifuging.

#### Kcr Peptide Enrichment

To enrich modified peptides, tryptic peptides dissolved in NETN buffer (100 mM NaCl, 1 mM EDTA, 50 mM *Tris-Hcl*, 0.5% NP-40, and pH 8.0) were incubated with prewashed antibody beads (Lot number 001, PTM BIO, Chicago, IL, United States) at 4°C overnight with gentle shaking. Then the beads were washed four times with NETN buffer and twice with H2O. The bound peptides were eluted from the beads with 0.1% trifluoroacetic acid. Finally, the eluted fractions were combined and vacuum-dried. For LC-MS/MS analysis, the resulting peptides were desalted with C18 Ziptips (Millipore, Bedford, MA, United States) according to the manufacturer’s instructions. The bio-material-based PTM enrichment (for phosphorylation) was as follows.

Peptide mixtures were first incubated with IMAC microsphere suspension with vibration in loading buffer (50% acetonitrile/6% trifluoroacetic acid). The IMAC microspheres with enriched phosphopeptides were collected by centrifugation, and the supernatant was removed. To remove nonspecifically adsorbed peptides, the IMAC microspheres were washed with 50% acetonitrile/6% trifluoroacetic acid and 30% acetonitrile/0.1% trifluoroacetic acid, sequentially. To elute the enriched phosphopeptides from the IMAC microspheres, elution buffer containing 10% NH4OH was added and the enriched phosphopeptides were eluted with vibration. The supernatant containing phosphopeptides was collected and lyophilized for LC-MS/MS analysis.

#### LC-MS/MS Analysis

The tryptic peptides were dissolved in 0.1% formic acid (solvent A), directly loaded onto a homemade reversed-phase analytical column (15-cm length and 75 μm i.d.). The gradient comprised an increase from 6 to 23% solvent B (0.1% formic acid in 98% acetonitrile) over 26 min, 23 to 35% in 8 min, and a climb to 80% in 3 min then holding at 80% for the last 3 min, all at a constant flow rate of 400 nl/min on an EASY-nLC 1,000 UPLC system. The peptides were subjected to an NSI source followed by tandem MS (MS/MS) in Q Exactive^TM^ Plus (Thermo) coupled online to the UPLC. The electrospray voltage applied was 2.0 kV. The m/z scan range was 350 to 1,800 for full scan, and intact peptides were detected in the Orbitrap at a resolution of 70,000. Peptides were then selected for MS/MS using an NCE setting of 28, and the fragments were detected in the Orbitrap at a resolution of 17,500. A data-dependent procedure alternated between one MS scan and 20 MS/MS scans with a 15.0-s dynamic exclusion. Automatic gain control (AGC) was set at 5E4. The fixed first mass was set as 100 m/z.

#### Database Search

The resulting MS/MS data were processed using a MaxQuant search engine (v.1.5.2.8). Tandem mass spectra were searched against the human UniProt database concatenated with the reverse decoy database. Trypsin/P was specified as a cleavage enzyme allowing up to four missing cleavages. The mass tolerance for precursor ions was set as 20 ppm in First search and 5 ppm in Main search, and the mass tolerance for fragment ions was set as 0.02 Da. Carbamidomethyl on Cys was specified as fixed modification, and acetylation modification and oxidation on Met were specified as variable modifications. FDR was adjusted to < 1%, and the minimum score for modified peptides was set > 40.

### Cell Lines and Cell Culture

Human 2BS and HDF fibroblasts were purchased from the National Cell Collection Center. HEK293 T cell lines come from our lab. All the cells were cultured in Dulbecco’s modified Eagle’s medium (DMEM, Invitrogen, United States) supplemented with 10% fetal bovine serum (FBS, Invitrogen, United States), 100 U/ml penicillin, and 100 mg/ml streptomycin and cultured in a humidified incubator at 37°C under 5% CO_2_ conditions.

### Establishment of a Cellular Senescence Model

Replicative senescence, bleomycin-induced premature senescence, irradiation-induced premature senescence, and RASV12-induced premature senescence were, respectively established according to our laboratory protocol.

### Animal Care and Ethics Statement

Four-week-old male Balb/c nu/nu mice and 8-week-old male C57BL/6 mice were purchased from Charles River (Wilmington, MA, United States). The mice were housed in a specific pathogen-free (SPF) environment with free access to food and water. The naturally aged male mice were fed on a normal diet for at least 24 months and then were used for identification of p16^INK4A^ staining and SA-gal staining. All experiments involving the handling of mice were approved by the animal ethics committee of the First Affiliated Hospital of Hunan Normal University. The human tissue samples were obtained with patients’ informed consent, and animal experiments and research on human tissues were approved by the Clinical Research Ethics Committee of the First Affiliated Hospital of Hunan Normal University.

### Coimmunoprecipitation

Total cells were lysed in BC100 buffer [20 mmol/l *Tris-Hcl* (pH 7.9), 100 mmol/l NaCl, 0.2% NP-40, and 20% glycerol] containing protease inhibitor cocktail (Sigma-Aldrich), 1 mmol/l dithiothreitol, and 1 mmol/l phenylmethylsulfonyl fluoride (Sigma-Aldrich). Whole lysates were incubated with 1 mg of mouse anti-SIRT7 or rabbit anti-PHF5A as described previously. For endogenous IP, protein was harvested, then lysed with BC100 buffer (100 mM NaCl, 20 mM Tris (pH 7.3), 20% glycerol, and 0.2% NP-40). Five percent of the cell lysates was collected as input. Cell extracts were incubated with 1 μg anti-PHF5A (Santa Cruz Biotechnology, Dallas, TX, United States) or normal mouse IgG (Santa Cruz Biotechnology) at 4°C overnight. Protein A/G agarose beads (Santa Cruz Biotechnology) were added to the lysates and incubated for 6 h at 4°C. Agarose beads were washed three times with BC100 buffer and boiled in the SDS sample buffer. Protein samples were separated by SDS-PAGE and immunoblotted with indicated antibodies.

### Immunoblots and Antibodies

The collected cells were lysed by lysis buffer containing a protease inhibitor cocktail (no. 05892791001, Roche, Basel, Switzerland) and a phosphatase inhibitor (P1092, Beyotime Biotechnology, Shanghai, China). The supernatants were mixed with protein loading buffer and separated by sodium dodecyl sulfate–polyacrylamide gel electrophoresis (SDS-PAGE; 10% sodium dodecyl sulfate), then electrotransferred onto a polyvinylidene difluoride (PVDF) membrane (Bio-Rad, Hercules, CA, United States). After blocking with 5% skimmed milk powder, we incubated the membranes overnight with primary antibodies against p16 (cat# ab189034; 1:1,000 dilution; Abcam, Cambridge, MA, United States), SIRT7 (cat# ab259968, Abcam, United States), PHF5A (cat#15554-1-AP, proteintech, China) and pan-kcr (cat#PTM502, PTMBIO, China), HA (cat# sc26183, Santa Cruz Biotechnology, United States), PHF5Ak25kcr (generated polyclonal antibodies against K25 crotonylation modifications by PTM Biolabs, Chicago, IL, United States), and GAPDH (cat# bs-0755R; 11,000 dilution; MBL, Nagoya, Japan) at 4°C overnight. After washing them three times, we then incubated the membranes with secondary antibodies (1:10,000 dilution; EarthOx Life Sciences, Millbrae, CA, United States) at room temperature for 1 h. Finally, we used an enhanced chemiluminescence (ECL, Millipore, United States) kit for visualization.

### CCK8 Assay and EdU Incorporation Assay

CCK8 assay was performed according to the previously described method (Yang et al., 2017). EdU incorporation assay was performed with an EdU incorporation assay kit according to the manufacturer’s instructions. All cells were examined using fluorescence microscopy with the appropriate filters. At least 500 cells were counted in randomly chosen fields from each culture well.

### Lentivirus Packaging

Lentivirus packaging was performed as described previously (Fu et al., 2019). HEK293T cells were cotransfected with lentiviral vectors psPAX2 and pMD2G. After transfection for 48 and 72 h, viral particles were collected, respectively by filtering through a 0.4-μm filter.

### Colony Formation Assay

Colony formation assay was conducted according to previously described methods (Yu A.Q. et al., 2020).

### Analysis of IHC

Immunohistochemistry analysis was performed as described previously (Yu A.Q. et al., 2020). Briefly, the formalin-fixed paraffin sections were deparaffinized, rehydrated, and pretreated with 3% hydrogen peroxide (H_2_O_2_) for 20 min to block endogenous peroxidase. After washing three times with TBST buffer, the antibody-binding epitopes of the antigens of specimens were retrieved by microwave boiling treatment at 98°C for 10 min, and the sections were then preincubated with 10% goat serum to block nonspecific binding sites. Rabbit anti-p16 (no. ab189034, Abcam, United States) and rabbit anti-SIRT7 (no. ab259968, Abcam, United States), diluted at ratios of 1:100, were used as the primary antibodies. After washing three times with TBST buffer, the specimens were incubated overnight with the primary antibodies at 4°C, followed by the addition of biotinylated anti-rabbit or anti-mouse secondary antibodies and streptavidin–horseradish peroxidase. Finally, we used 3,3′-diaminobenzidine (DAB) as a chromogen, and hematoxylin and eosin (HE) for counterstaining.

### Senescence-Associated Beta-Galactosidase Staining

Senescence-associated–β-gal activity assay was performed as described previously (Wang et al., 2017).

### RT-qPCR

Total RNA was extracted using TRIzol reagent (no. 15596018; Life Technologies, Eugene, OR, United States). Complementary DNA (cDNA) was synthesized under a template of extracted total RNA using a ReverTra Ace^®^ qPCR RT kit (no. FSQ101, Toyobo, Osaka, Japan) according to the manufacturer’s instructions. The cDNA samples were amplified using a SYBR^®^ Green Realtime PCR Master Mix (no. QPK201, Toyobo, Japan) in the 7,500 Real-Time PCR System (Applied Biosystems, Foster City, CA, United States). Primers used in this study are provided in Table 1.

**TABLE 1:** Primers used in this study.

List of oligonucleotides sequences (F, forward and R, reverse)	5′– > 3′
**Primers for RT-qPCR**	
SIRT7-F	ACGCCAAATACTTGGTCGTCT
SIRT7-R	AGCACTAACGCTTCTCCCTTT
CDK2-F	CCAGGAGTTACTTCTATGCCTGA
CDK2-R	TTCATCCAGGGGAGGTACAAC
GAPDH-F	CTGGGCTACACTGAGCACC
GAPDH-R	AAGTGGTCGTTGAGGGCAATG
**Primers for SIRT7 interference (shSIRT7)**	
sh-scramble	TTCTCCGAACGTGTCACGT
sh-SIRT7-1	GAACGGAACTCGGGTTATT
sh-SIRT7-2	TAGCCATTTGTCCTTGAGGAA
**Primers for SIRT7 overexpression (SIRT7 OV)**	
SIRT7 OV-F	GGAATTCAATGGCAGCCGGGGGTCTGAGC
SIRT7 OV-R	GCTCTAGACGTCACTTTCTTCCTTTTTGTG
**Primers for SIRT7 gst-pulldown**	
SIRT7-F (1–267)	GGAATTCAATGGCAGCCGGGGGTCTGAGC
SIRT7-R (1–267)	GCTCTAGACCTTCCCCCGCAGCTCC
SIRT7-F (268–993)	GGAATTCAATGCGGGAGCTGGCCAGCGC
SIRT7-R (268–993)	GCTCTAGGGGATCTCCAAGCCCAGC
SIRT7-F (994–1200)	GGAATTCAATGGCCTATAGCAGGTGGCAG
SIRT7-R (994–1200)	GCTCTAGACGTCACTTTCTTCCTTTTTGTG
SIRT7-F (1–1200)	GGAATTCAATGGCAGCCGGGGGTCTGAGC
SIRT7-R (1–1200)	GCTCTAGACGTCACTTTCTTCCTTTTTGTG

### 2BS Transplantation Assays

1 × 10^7^ 2BS transduced with a lentivirus expressing Luc were injected into the abdominal subcutaneous of nude mice (6 to 8 weeks, male). Then, 0, 2, and 4 days after transplantation, mice were treated with D-luciferin substrate and imaged with an IVIS Spectrum imaging system.

### Statistical Analysis

The quantitative data from three independent biological repeats were presented as the means ± standard deviations (SDs). The statistical analyses were performed using 25.0 SPSS software, and statistical significance between groups was determined by Student’s *t*-tests or analysis of variance (ANOVA). A value of *p* < 0.05 is considered statistically significant. ^∗^ denotes *p* < 0.05, ^∗∗^ denotes *p* < 0.01, and ^∗∗∗^ denotes *p* < 0.001. N.S. denotes no significance.

## Data Availability Statement

The datasets presented in this study can be found in online repositories. The names of the repository/repositories and accession number(s) can be found below: PRIDE database, accession no. PXD027232.

## Ethics Statement

The studies involving human participants were reviewed and approved by the Ethics Committee of Hunan Normal University. The patients/participants provided their written informed consent to participate in this study.

## Author Contributions

ZW, LT, and XH conceived the idea. AY and ZW discussed the data and wrote the manuscript. AY, SJ, LY, HM, and JW designed and performed the cellular and molecular experiments, and analyzed the data. AY, SJ, and YH performed animal experiments and collected the data. AY, SJ, YH, LY, and HM established the cellular senescence model. All authors contributed to the article and approved the submitted version.

## Conflict of Interest

The authors declare that the research was conducted in the absence of any commercial or financial relationships that could be construed as a potential conflict of interest.

## Publisher’s Note

All claims expressed in this article are solely those of the authors and do not necessarily represent those of their affiliated organizations, or those of the publisher, the editors and the reviewers. Any product that may be evaluated in this article, or claim that may be made by its manufacturer, is not guaranteed or endorsed by the publisher.
