# Supplementary material for: SIRT7-Induced PHF5A Decrotonylation Regulates Aging Progress Through Alternative Splicing-Mediated Downregulation of CDK2
Source: Front Cell Dev Biol. 2021 Sep 17;9:710479. doi: 10.3389/fcell.2021.710479 (PMC8484718; doi:10.3389/fcell.2021.710479)
Supplement: Supplementary file 1 [file Data_Sheet_1.DOCX]

**SIRT7-induced PHF5A de-crotonylation regulates ageing progress** **through alternative splicing-mediated downregulation of CDK2**

Figure S1 The **expression profile of Kcr proteins in senescent fibroblasts**


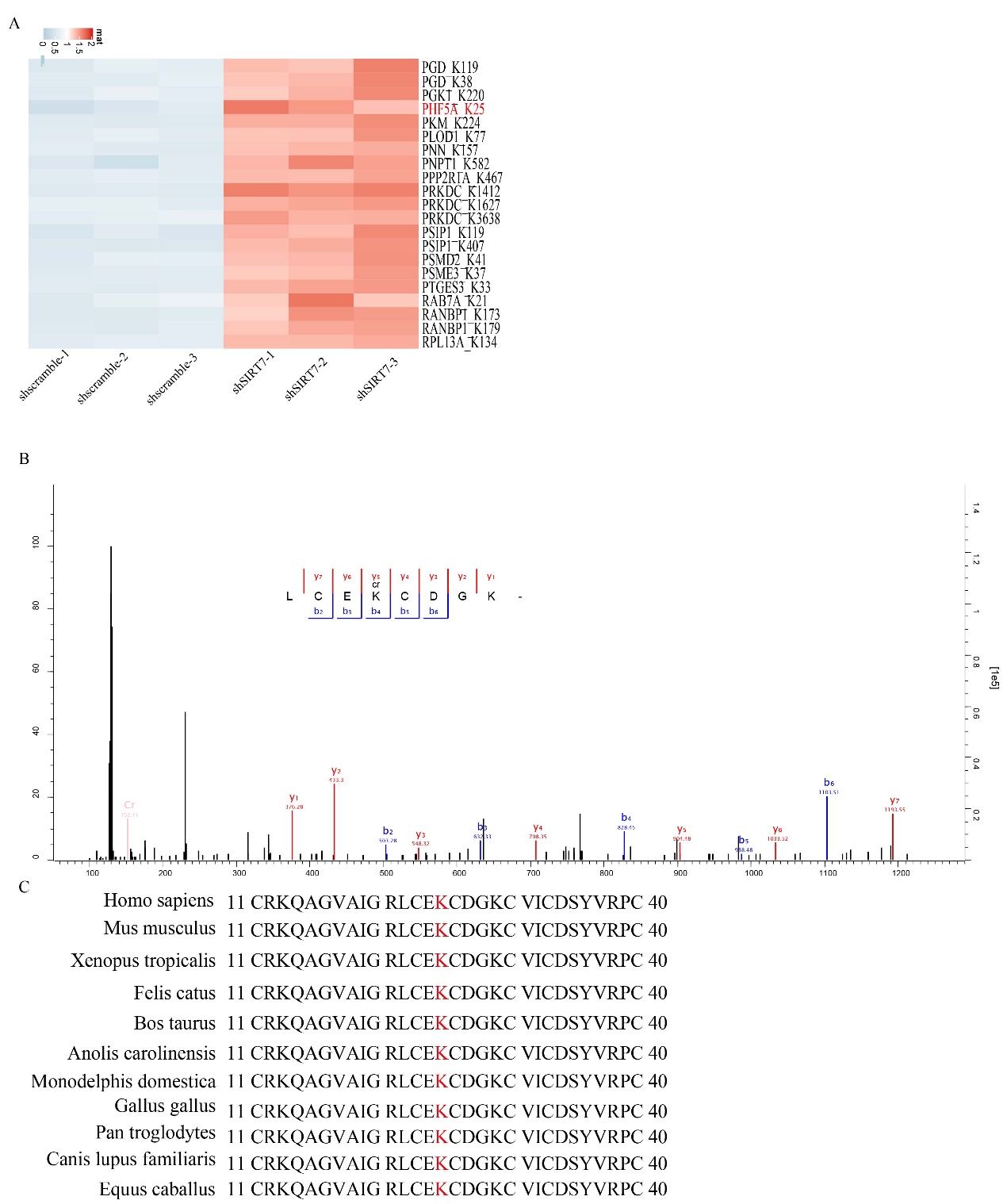


(A). The heatmap showing partial upregulated Kcr proteins; (B). Identified crotonylation site of PHF5A at k25; (C) PHF5A K25 is evolutionarily conserved. The sequences of PHF5A in eleven species were aligned. Lysine 25 of PHF5A was highlighted in red

Figure S2 **Detection of the CDK2 mRNA expression in both PHF5A wild type and PHF5A K25R fibroblasts upon SIRT7 knocking down and overexpression**


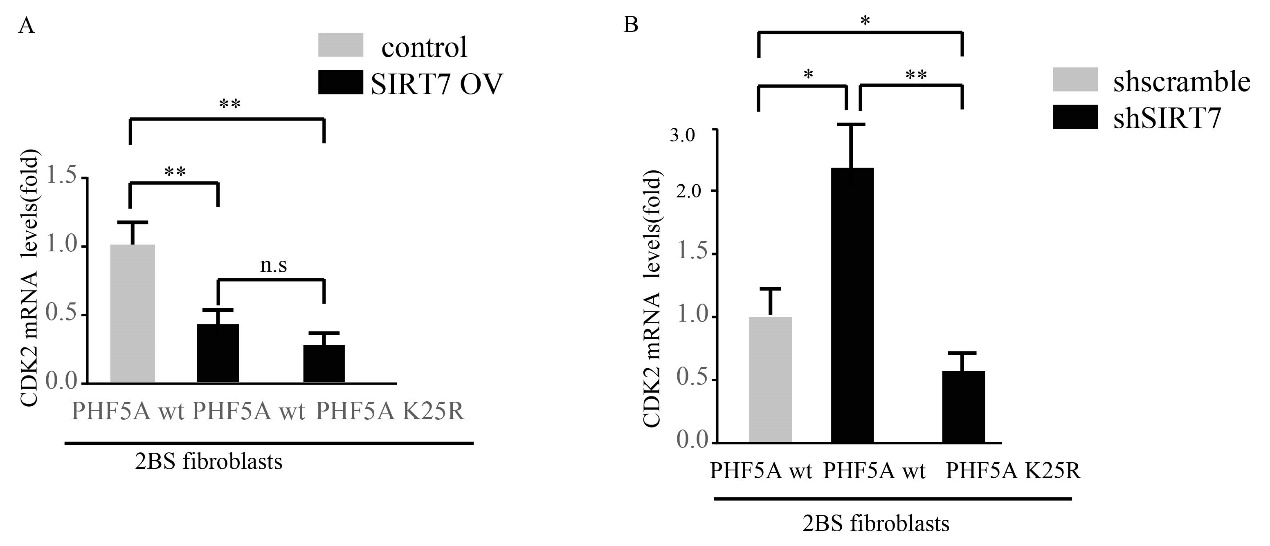


qRT-PCR detection of the CDK2 mRNA expression in both PHF5A wild type and PHF5A K25R fibroblasts upon SIRT7 knocking down(A) and overexpression(B).

Figure S3 **Detection of SIRT7 protein level in replicative and premature senescent fibroblasts**


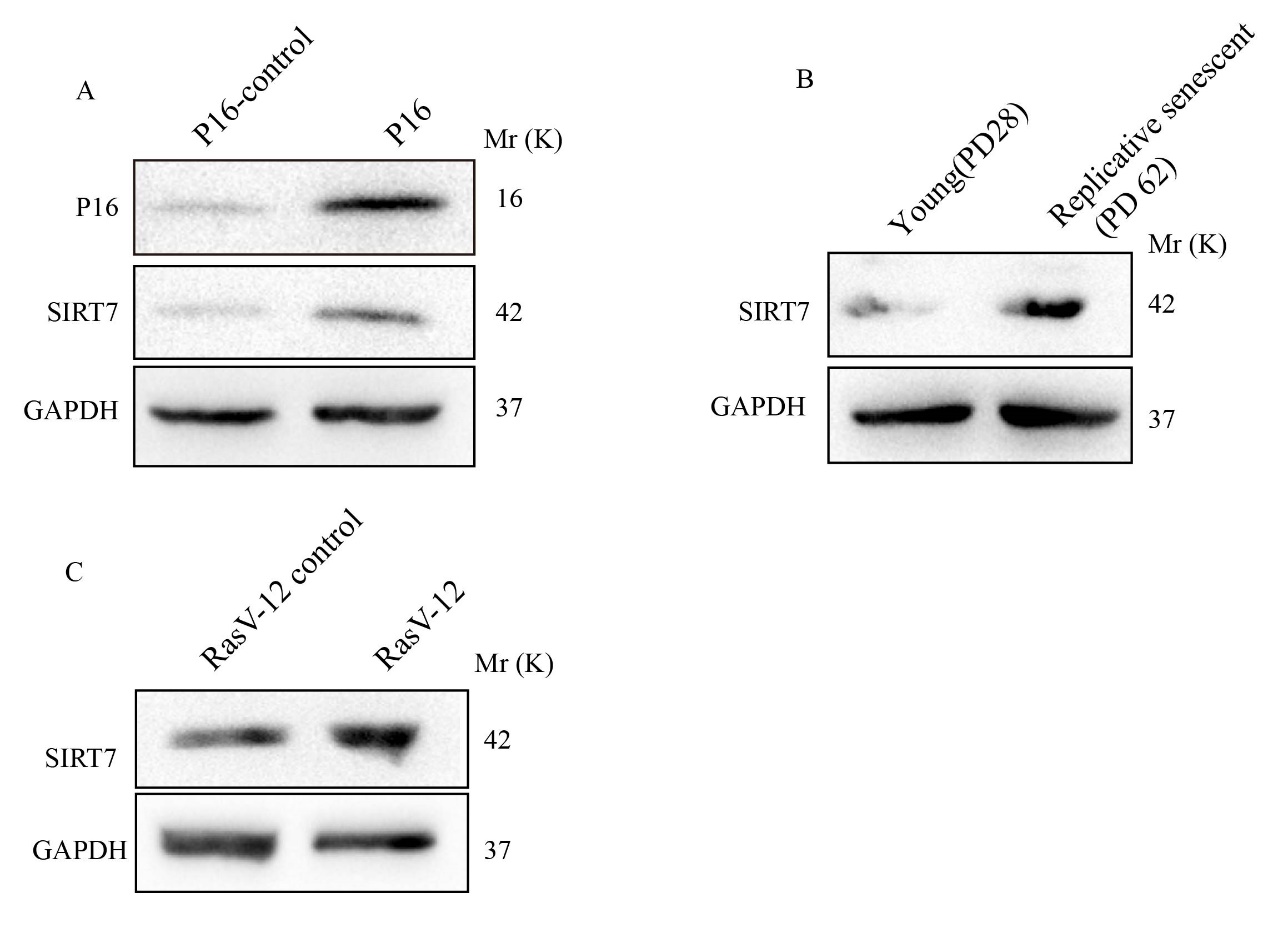


Immunoblot detection of SIRT7 protein level in p16-induced premature senescent fibroblasts(A), replicative senescent fibroblasts(B) and RasV12-induced premature senescent fibroblasts(C).
